# Supplementary material for: Hetero-oligomerization of TDP-43 carboxy-terminal fragments with cellular proteins contributes to proteotoxicity
Source: Commun Biol. 2024 Jun 20;7:743. doi: 10.1038/s42003-024-06410-3 (PMC11190292; doi:10.1038/s42003-024-06410-3)

Supplemental Figures

Hetero-oligomerization of TDP-43 carboxy-terminal fragments with cellular proteins contributes to proteotoxicity

Akira Kitamura[1,2,3†*], Ai Fujimoto[1†], Rei Kawashima[3], Yidan Lyu[3], Kotetsu Sasaki[1], Yuta Hamada[1], Kanami Moriya[3], Ayumi Kurata[3], Kazuho Takahashi[3], Reneé Brielmann[4], Laura C. Bott[4], Richard I. Morimoto[4], Masataka Kinjo[3]

1 Laboratory of Cellular and Molecular Sciences, Faculty of Advanced Life Science, Hokkaido University, N21W11, Kita-ku, Sapporo, Japan 001-0021.
2 PRIME, Japan Agency for Medical Research and Development, Chiyoda-ku, Tokyo, Japan 100-0004.
3 Laboratory of Molecular Cell Dynamics, Faculty of Advanced Life Science, Hokkaido University, N21W11, Kita-ku, Sapporo, Japan 001-0021.
4 Department of Molecular and Cell Biology and Biochemistry, Rice Institute for Biomedical Research, Northwestern University, Evanston, IL, USA 60208.
* To whom correspondence should be addressed: akita@sci.hokudai.ac.jp
† Equally contributed

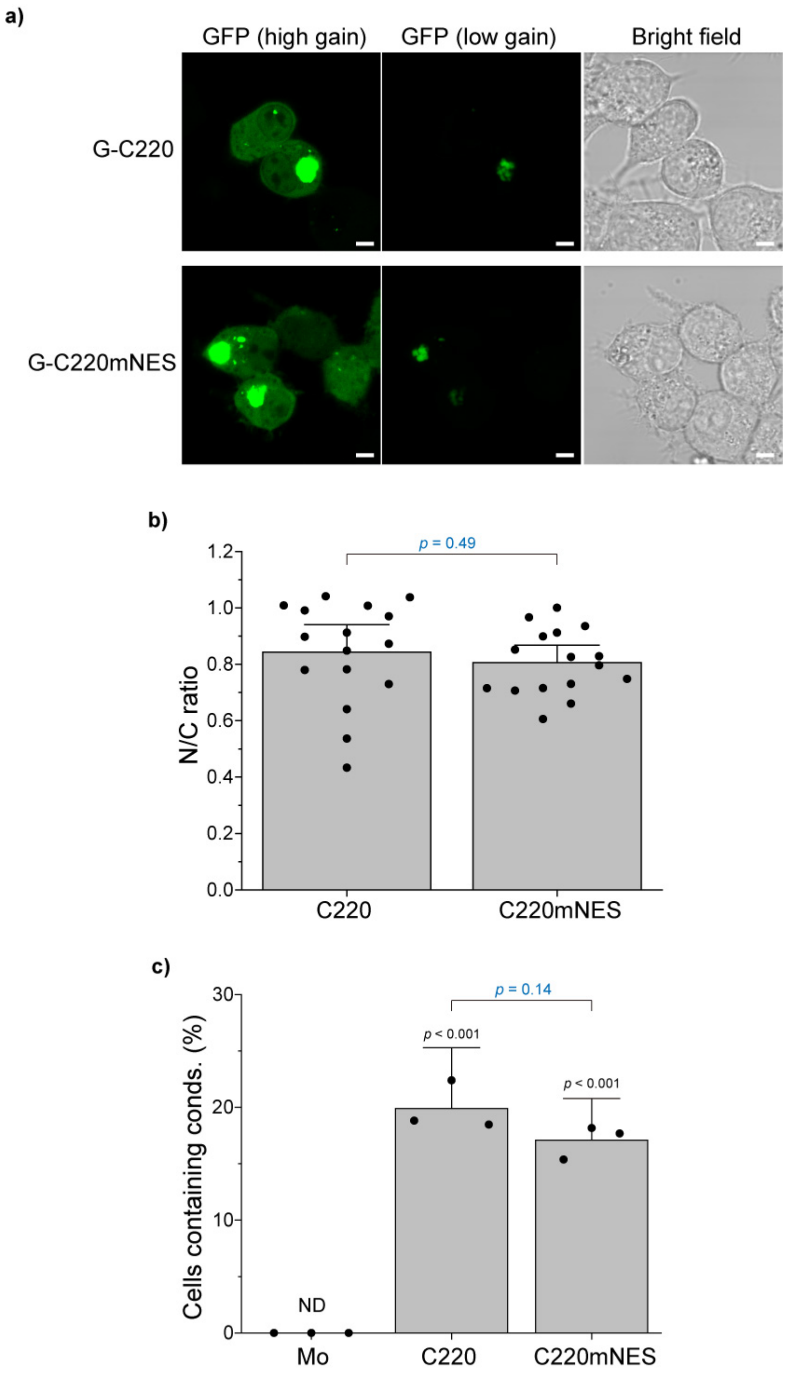

Supplemental Figure 1. Subcellular distribution and cytoplasmic condensation of C220 carrying mutations for nuclear exporting signal sequence in Neuro2a cells

a) Confocal images of Neuro2a cells expressing GFP-tagged C220 (G-C220) and C220 mutations for nuclear exporting signal sequence (NES) (G-C220mNES). The left and middle fluorescence images were acquired from the same field of view using either high or low gain of the detector. Bar = 5 µm. **b)** Dots and bar graphs indicate relative fluorescence intensity in the nucleoplasm to that in the cytoplasm (N/C ratio). **c)** Dots and bar graphs indicate the population of the cells containing cytoplasmic condensates (mean ± 95% CI; n = 3 independent experiments). ND: Not determined; *p*-values above the lines: one-way ANOVA with Tukey's test.

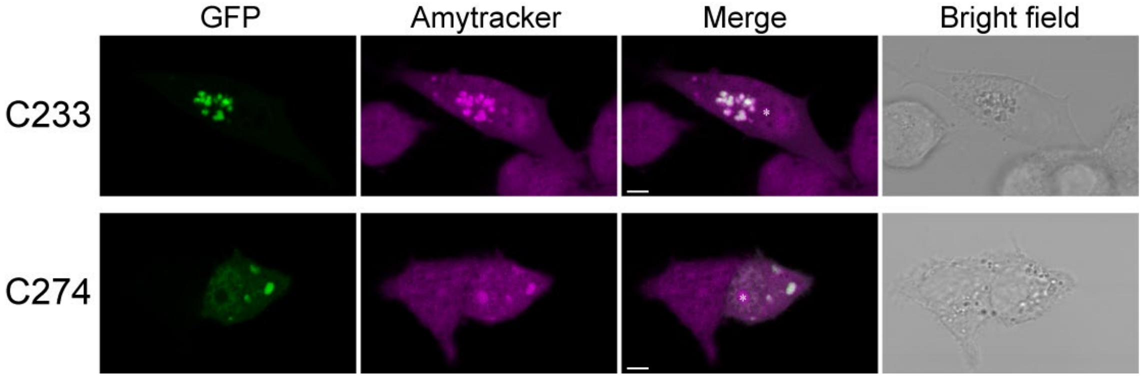

Supplemental Figure 2. Amytracker staining of the C233 and C274 condensates in Neuro2a cells
Confocal images of GFP-tagged C233 and C274-expressing Neuro2a cells stained with a fluorescent tracer for amyloid, Amytracker. The asterisk in the images represents the nucleolus. Bars = 5 μm.

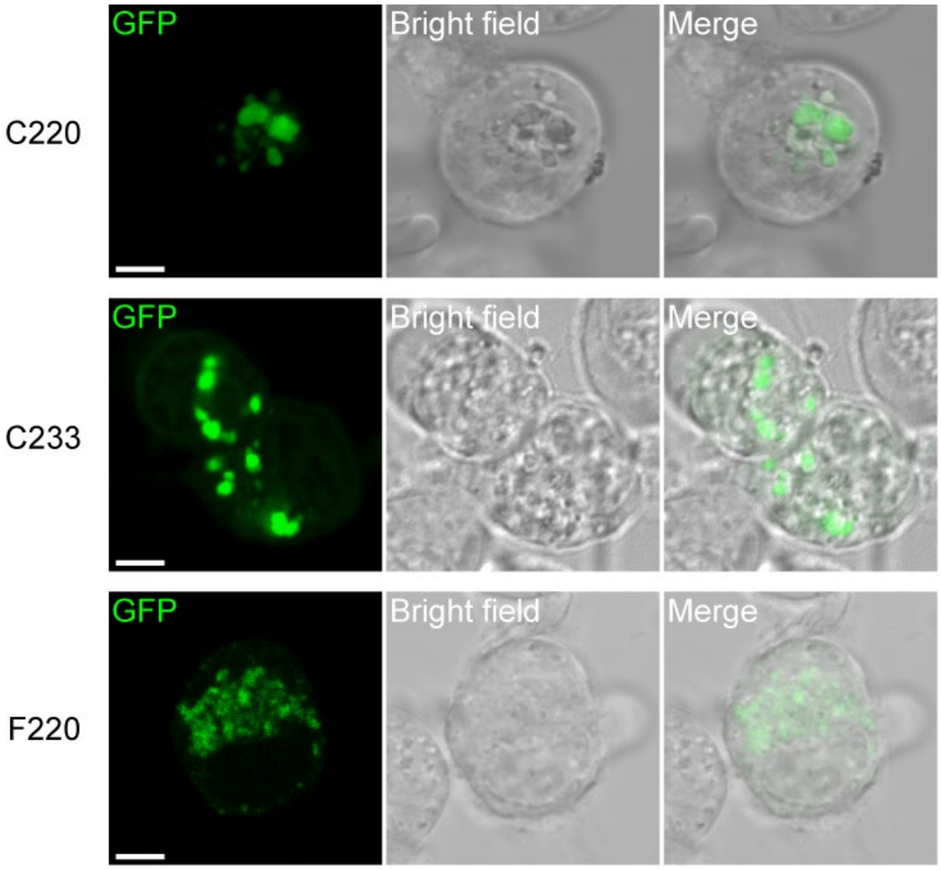

Supplemental Figure 3. Confocal fluorescent images of Neuro2a cells expressing C220, C233, and F220 tagged with GFP after the 1,6-Hexanediol treatment.

The confocal fluorescence images of cells containing cytoplasmic condensates were treated with 1,6-hexanediol were represented. Bars = 5 μm.

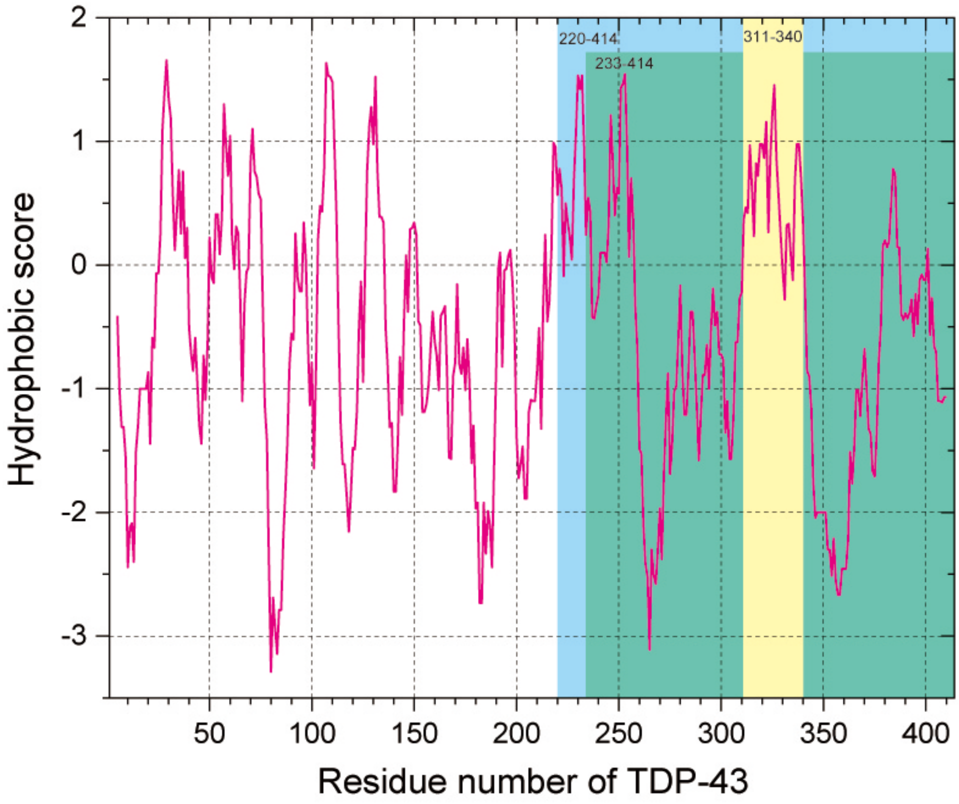

Amino acid	Score
Ile	4.5
Val	4.2
Leu	3.8
Phe	2.8
Cys	2.5
Met	1.9
Ala	1.8
Gly	-0.4
Thr	-0.7
Ser	-0.8
Trp	-0.9
Tyr	-1.3
Pro	-1.6
His	-3.2
Asn	-3.5
Asp	-3.5
Gln	-3.5
Glu	-3.5
Lys	-3.9
Arg	-4.5

Supplemental Figure 4. Hydropathy plot of human TDP-43 amino acid sequence

Left: The hydropathy plot was calculated using the Expasy Web server [Kyte J. & Doolittle R.F., ***J. Mol. Biol.***, 157, 105-132 (1982)]. The CTF regions of C220 and C233 represented a cyan and green area, respectively. A highly hydrophobic region in GRR was shown in a yellow area. *Right*: The hydrophobicity scores of each amino acid are represented (Red to blue: high to low hydrophobicity).

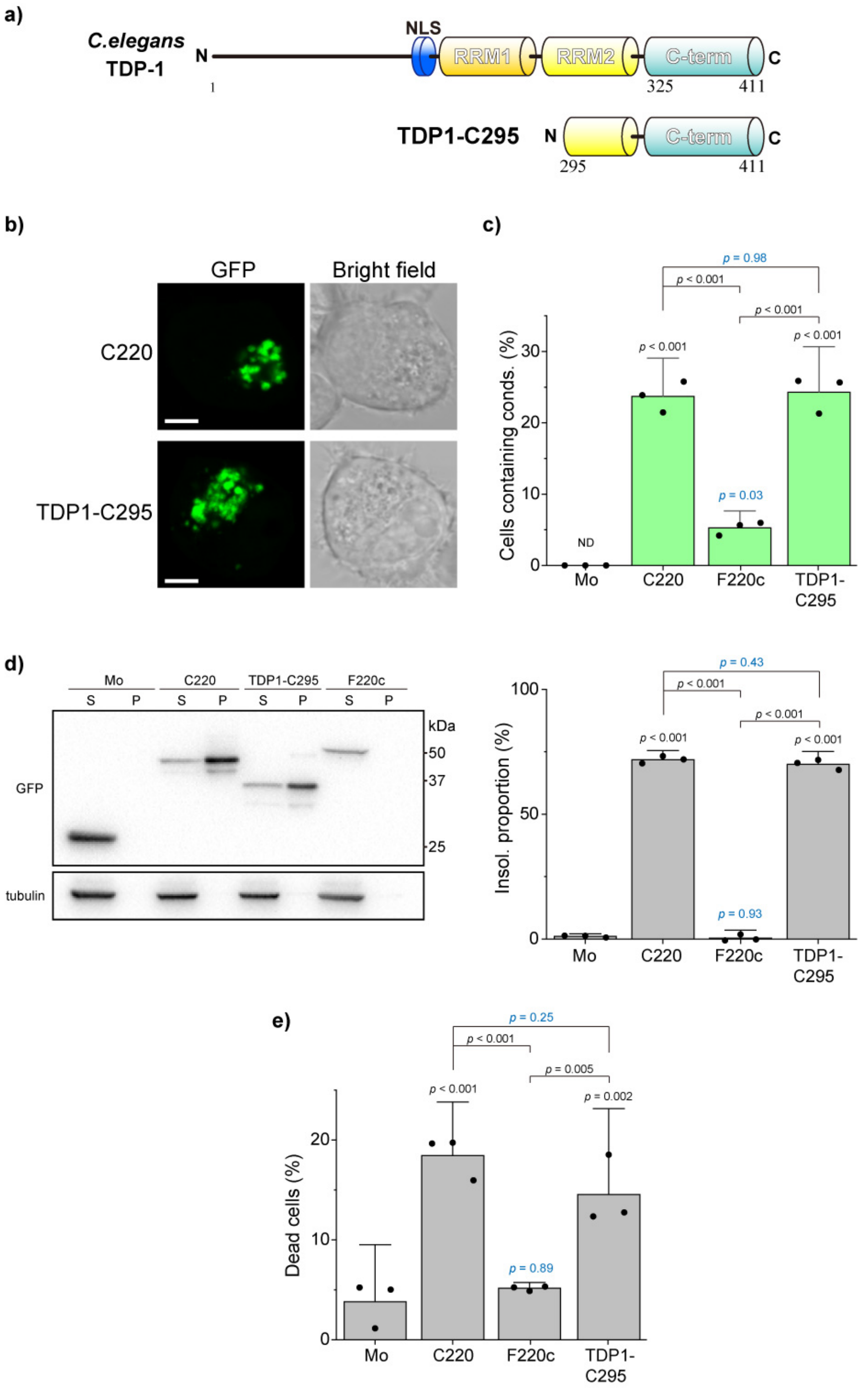

Supplemental Figure 5. Formation of the cytoplasmic condensates and toxicity of *Caenorhabditis elegans* TDP-1 carboxy terminal fragment in Neuro2a cells

a) Primary structures and abbreviation of *Caenorhabditis elegans* TDP-1 and its carboxy-terminal fragment (TDP1-C295: 295–411 amino acids) that corresponds to human C220/TDP25. The RRM and C-term denote the RNA/DNA-recognition motif and the C-terminal region, respectively. **b)** Confocal fluorescence and bright field microscopic images of Neuro2a cells expressing GFP-tagged C220 and TDP1-C295. Bars = 5 μm. **c)** The population of Neuro2a cells containing cytoplasmic condensates of GFP-tagged C220, tRRM2 (F220c), TDP1-C295, and GFP monomers (Mo). Bars: mean + 95% CI; Dots: raw values (n = 3 independent experiments). **d)** Western blot stained using an anti-GFP and anti-

α-tubulin antibody of cell lysates: 1% SDS-soluble (S) and insoluble (P) (*left*). The quantification of the abundance of TDP-43 CTFs, TDP1-C295, and GFP monomers (Mo) in the insoluble fraction. The abundance shows the normalized band intensity in the P fraction to total (S+P) fraction (n = 3 independent experiments) (bottom). **e)** Population of dead cells expressing TDP-43 CTFs and TDP1-C295 tagged with GFP and GFP monomers (Mo) using a propidium iodide exclusion test. Bars: mean and 95% CI (n = 3 independent experiments). **c)**, **d)**, and **e)** *p*-values above the bars and lines: one-way ANOVA with Tukey's test compared to GFP monomers as control and comparison between lines, respectively. ND: not determined.

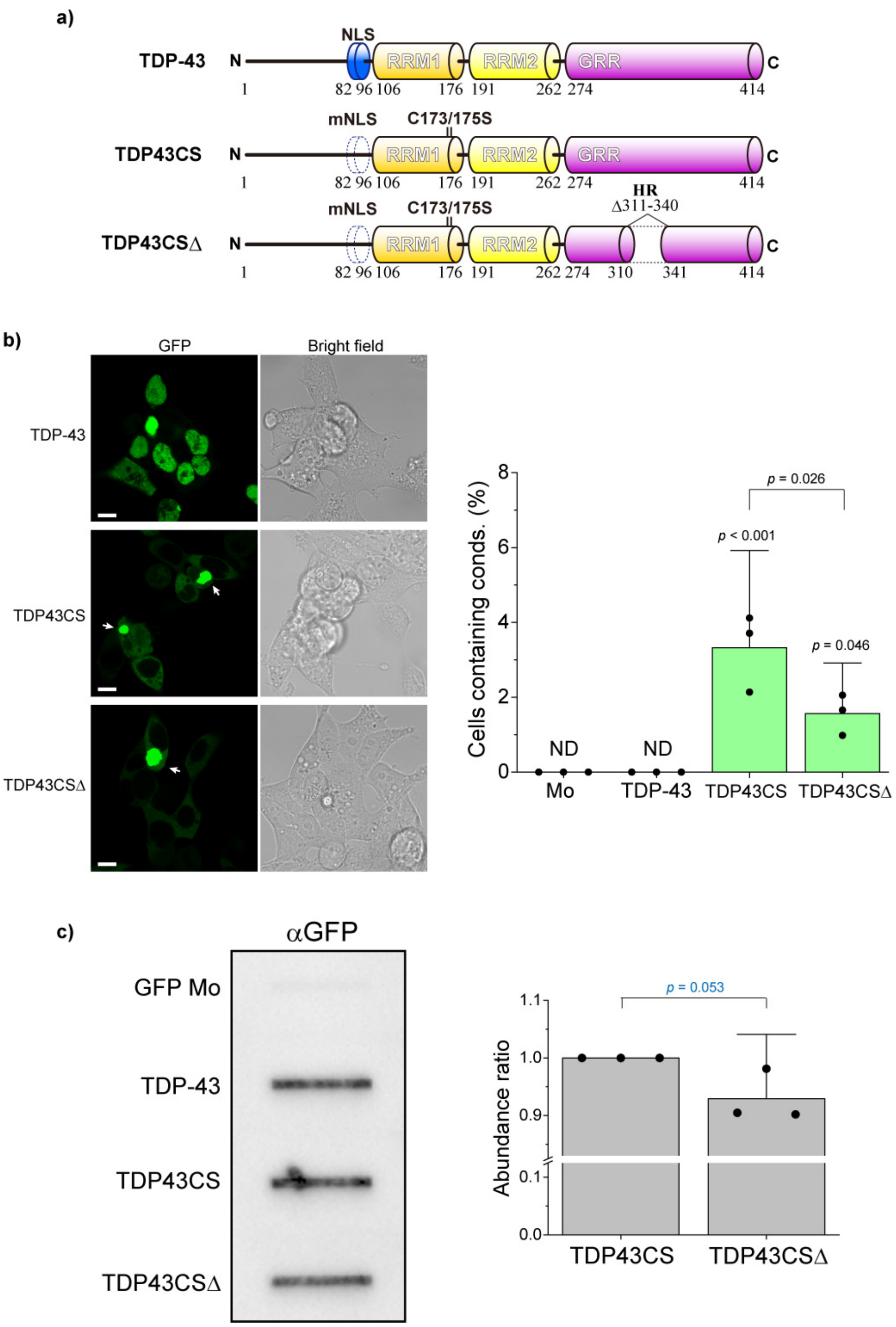

Supplemental Figure 6. Formation of the cytoplasmic condensates of full-length TDP-43 in Neuro2a cells

a) Primary structures and abbreviation of human TDP-43 and its mutants. The RRM and GRR denote the RNA/DNA-recognition motif and the glycine-rich region, respectively. TDP43CS carries mutations in nuclear localization signal sequence (mNLS) and two substitutions of 173th and 175th cysteine to serine residues. TDP43CSΔ lacks hydrophobic region (HR) in GRR. All proteins are tagged with monomeric GFP at the C-terminus. **b)** Confocal fluorescence and bright field microscopic images of Neuro2a cells expressing GFP-tagged TDP-43, TDP43CS, and TDP43CSΔ.

Bars = 10 μm. **c)** The population of Neuro2a cells containing cytoplasmic condensates of GFP-tagged TDP-43, TDP43CS, TDP43CSΔ, and GFP monomers (Mo). Bars: mean + 95% CI; Dots: raw values (n = 3 independent experiments). **d)** Filter retardation assay followed by staining using an anti-GFP antibody of cell lysates (*left*). The quantification of the abundance ratio of TDP43CS and TDP43CSΔ retarded on the membrane (n = 3 independent experiments) (*right*). *p*-values above the bars and lines: one-way ANOVA with Tukey's test compared to GFP monomers as control and comparison between lines, respectively. ND: not determined.

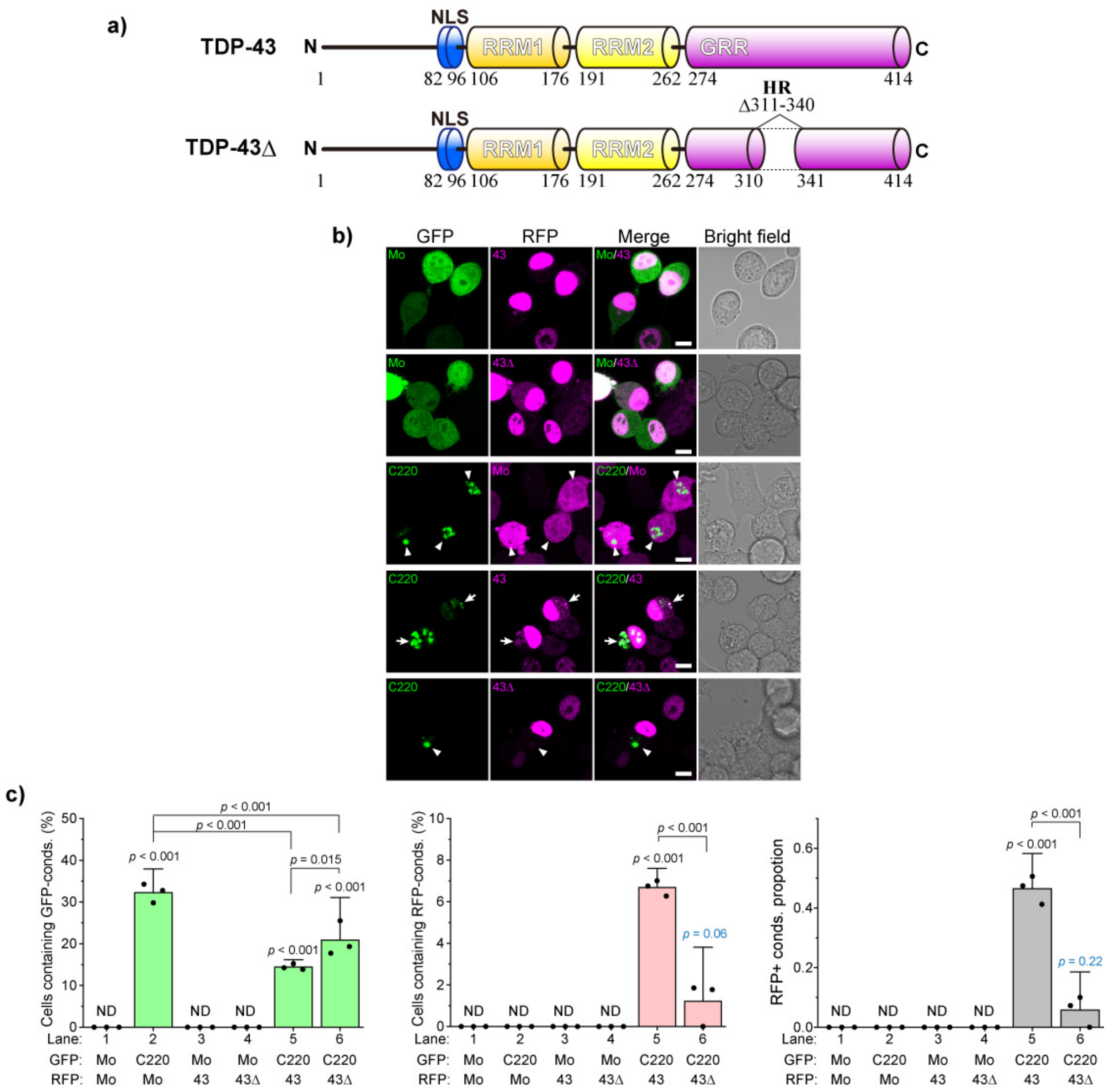

Supplemental Figure 7. The role of hydrophobic region in TDP-43 glycine-rich region during co-condensation between C220 and full-length TDP-43.

a) Domain structure and abbreviation of human full-length TDP-43 and that lacking 311-340 region (TDP43Δ). HR and GRR denote a hydrophobic region and a glycine-rich region, respectively. **b)** Confocal fluorescence and bright field microscopic images of Neuro2a cells expressing GFP-tagged C220 and RFP-tagged TDP-43 (43) or TDP43Δ (43Δ). Mo indicates GFP or RFP monomers. White arrowheads represent the position of green fluorescence-positive but red fluorescence-negative condensates in the cytoplasm. White arrows represent both green and red fluorescence-positive condensates in the cytoplasm. Bars = 5 μm. **c)** *left*: The population of Neuro2a cells that contain GFP-positive condensates (conds) in the cytoplasm. *Middle*: The population of Neuro2a cells containing RFP-positive condensates in the cytoplasm. *Right*: The ratio of RFP-positive condensates to GFP-positive ones. ND: Not determined; N/A: Not applicable; *p*-values above the lines: one-way ANOVA with Tukey's test (n = 3 independent experiments).

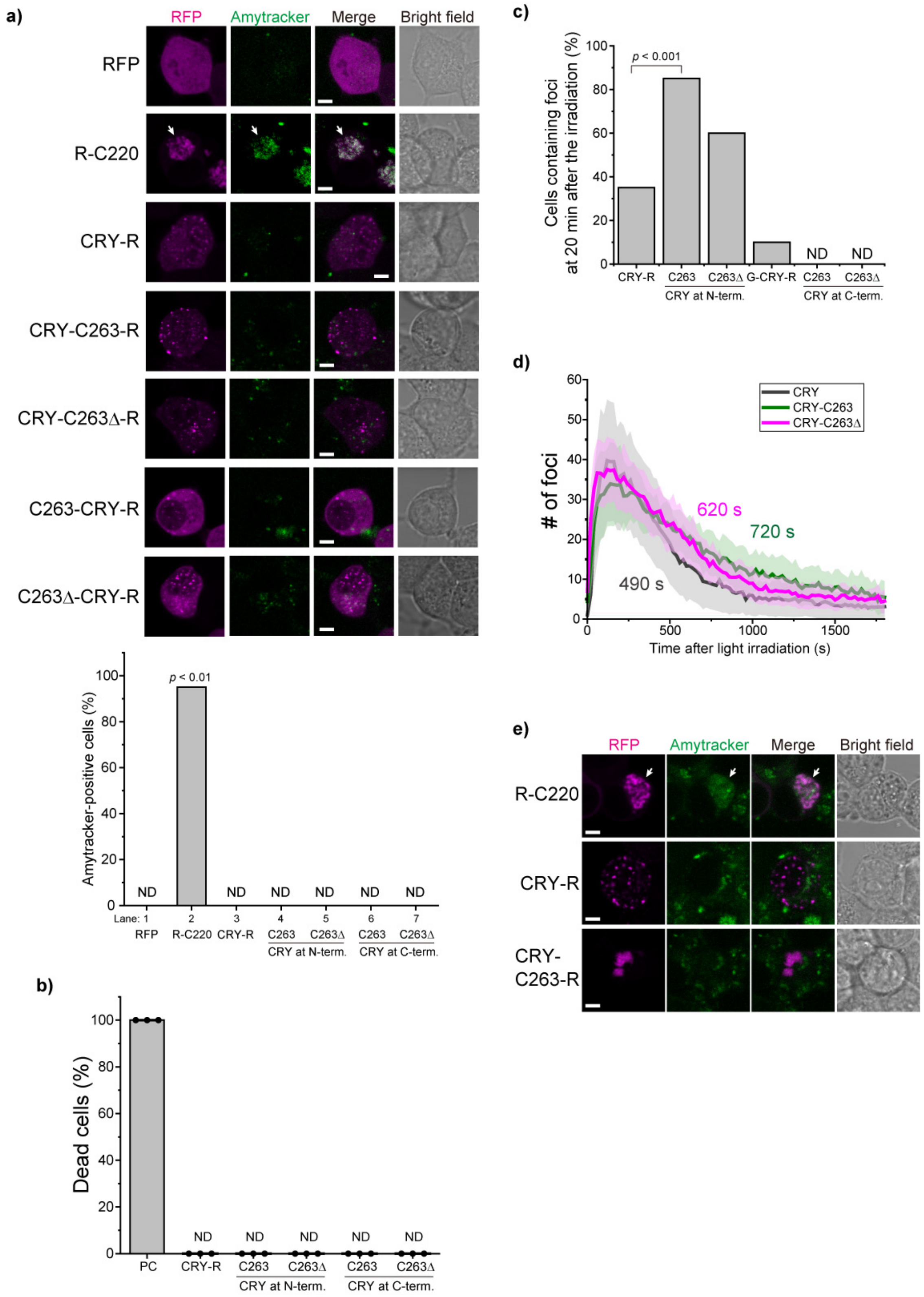

Supplemental Figure 8. Fate and consequence of light-induced condensates of TDP-43 carboxy-terminal fragments.

a) *Top*: Fluorescence images of Amytracker 520-stained Neuro2a cells expressing TDP-43 CTFs: C220, C220Δ, C263, and C263Δ tagged with a cryptochrome-derived oligomerization inducer, CRY2olig

(CRY), RFP monomers (RFP), and RFP-tagged C220 at 30 min after the 488 nm-light irradiation. The order of the hyphens before and after indicates the N/C-terminal side of the CRY tag. White arrowheads represent Amytracker-positive condensates. Bars = 5 μm. *Bottom*: Population of cells that form foci after the blue-light irradiation obtained from all measured cells. ND: Not determined; *p*-values above the bars: hypothesis test for the difference in the population proportions compared to RFP monomers. **b)** Population of dead cells using a DRAQ7 dye exclusion test. PC denotes ethanol-treated cells as a positive control. Bars: mean and 95% CI (n = 3 independent experiments). **c)** Population of cells harboring foci at 20 min after the light irradiation. *p*-values were obtained by hypothesis test for the difference in the population proportions compared to CRY-R. **d)** Time-course of the number of light-induced foci of CRY, CRY-C263, and CRY-C263Δ in a cell after turning off the blue light (dark gray, green, and magenta, respectively). The thick solid line represents the mean, and the shaded area in a lighter color indicates ± 95% CI (n = 20 independent cells). Inset numbers indicate mean half-decreasing times after their maximum peak. **e)** Amytracker-stained Neuro2a cells harboring TDP25, CRY, and CRY-C263 foci that remained after 8 hours of blue light irradiation. White arrowheads indicate Amytracker-positive foci. Bars = 5 μm.

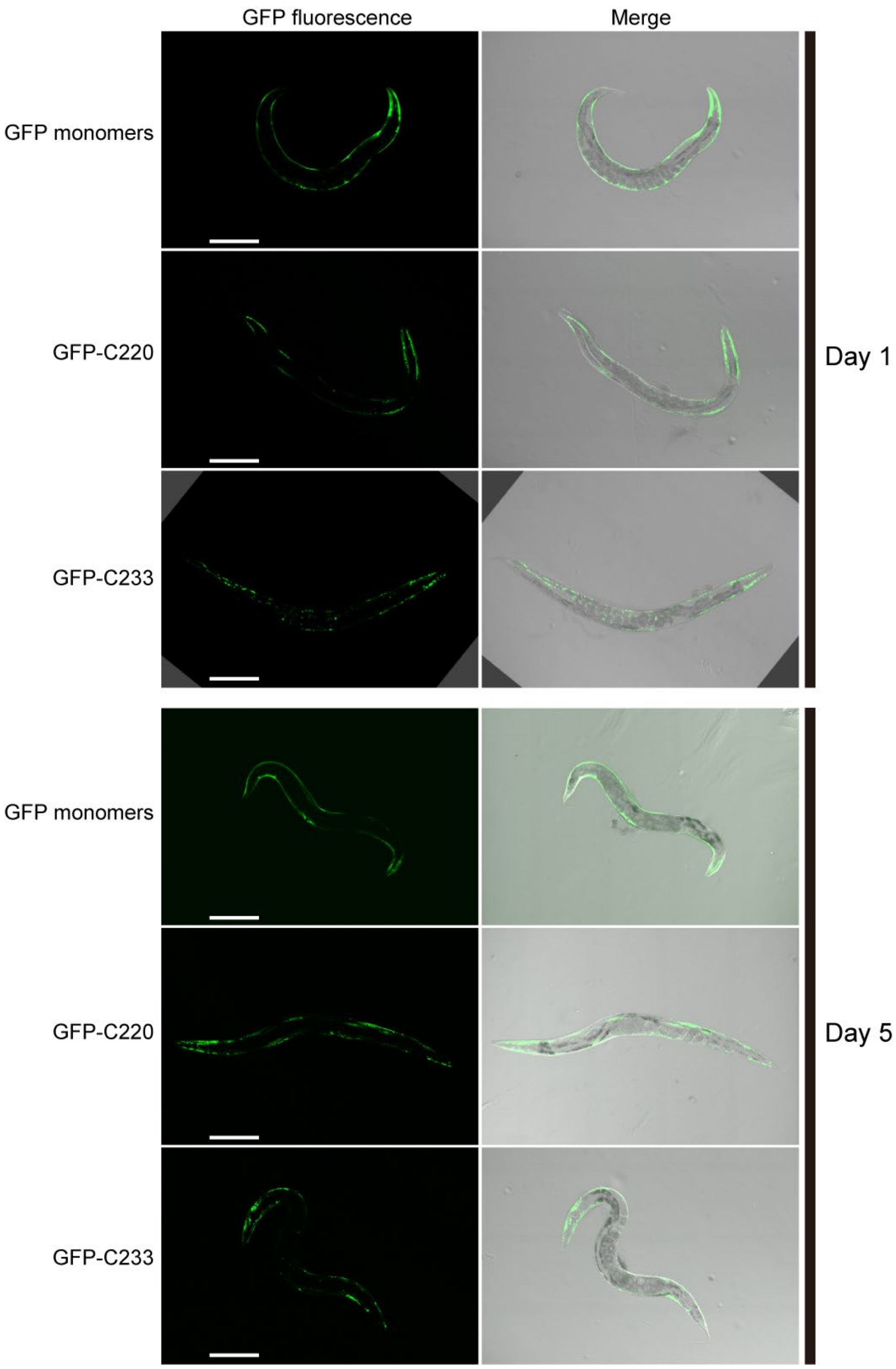

Supplemental Figure 9. Confocal fluorescence images of nematodes expressing GFP, GFP-C220, and GFP-C233.

Total view of the nematodes expressing GFP-C220, GFP-C233, and GFP monomers on 4 or 8 days after synchronization by bleaching (Day 1 and 5 of adulthood, respectively) shown in Figure 8A. Bars = 0.2 mm.

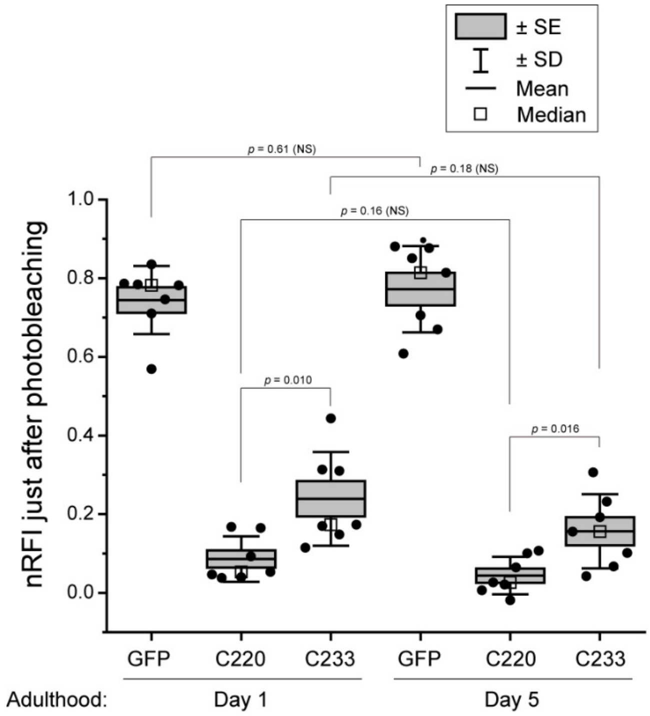

Supplemental Figure 10. Comparison of normalized relative fluorescence intensities (nRFI) just after photobleaching of C220 and C233 tagged with GFP in *C. elegans*.

Box and bars with dots plot of nRFI of GFP monomers, GFP-C220, and GFP-C233 just after photobleaching (t = 0 s in Figure 9A). *p*-values above the lines: one-way ANOVA with Tukey's test (n =7 independent aminals); NS indicates $p > 0.05$.

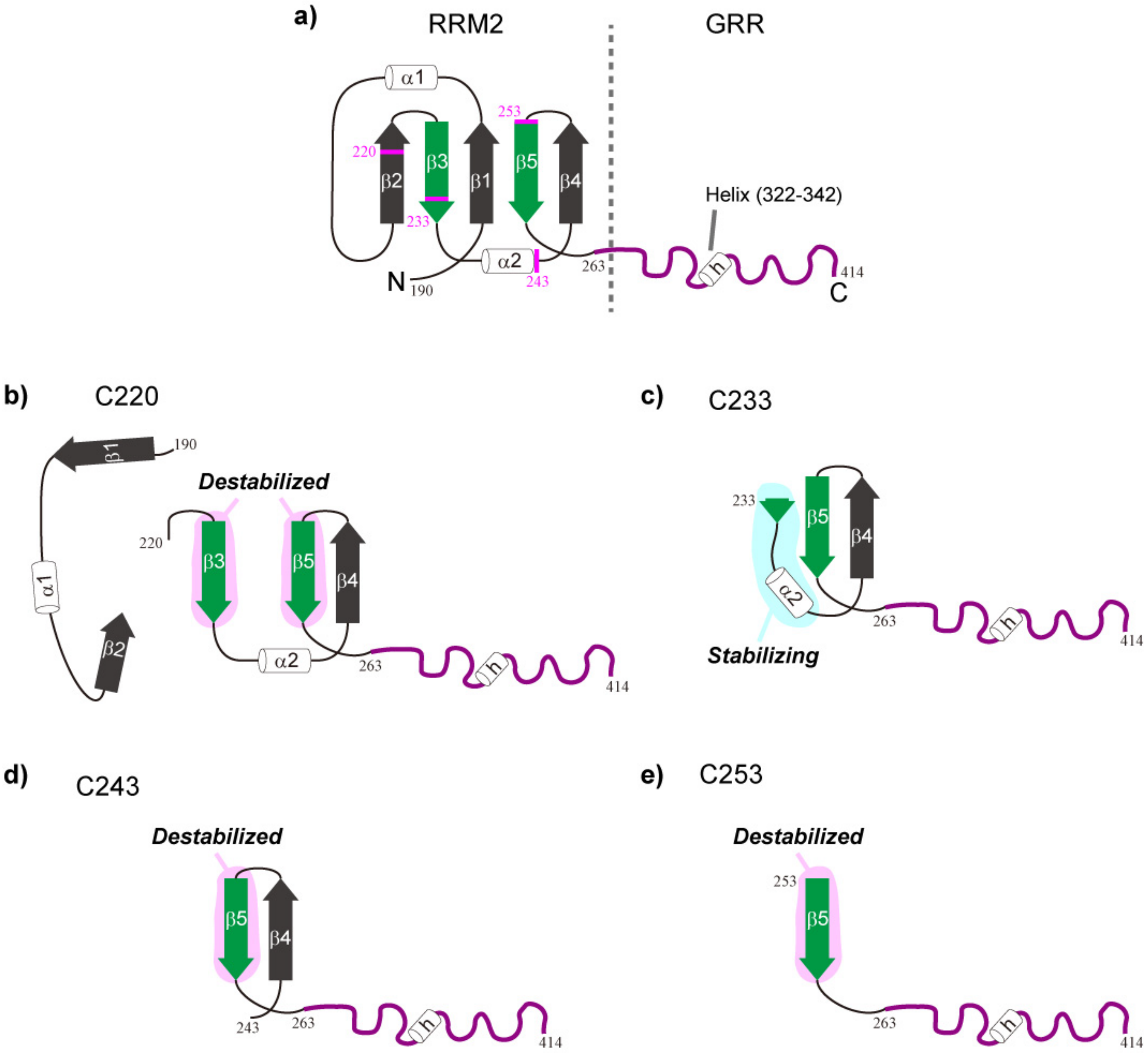

Supplemental Figure 11. Illustration of the secondary structure of intact RRM2 and GRR of TDP-43 and the cleavage positions of C220, C233, C243, and C253

(A–E) α1 and α2 indicate two α-helixes and β1–5 indicate five β-strands in RRM2 of TDP-43 observed in PDB#3D2W. The h means α-helical structure in GRR of TDP-43 observed in PDB#2N3X [1]. Structure of intact RRM2 and GRR (A), C220/TDP25 and the cleaved fragment of RRM2 (B), C233 (C), C243 (D), and C253 (E).

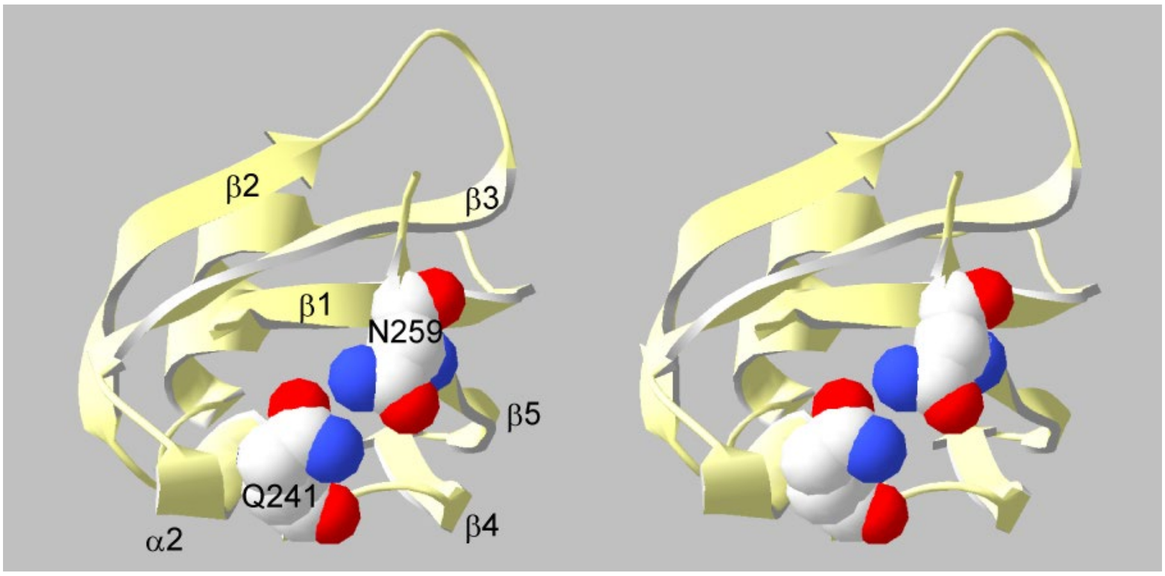

Supplemental Figure 12. Stereoview of the electrostatic interaction of the amino acid side chain (Q241 and N259) in RRM2

α1 and α2 indicate two α-helixes and β1–5 indicate five β-strands in RRM2 of TDP-43 observed in PDB#3D2W. The balls indicate the atoms of the side chain of Q241 and N259 (red and blue balls represent atoms having positive and negative charges, respectively).

Supplemental Figure 13. Uncropped and unedited blot images

Figure 1d. GFP blot

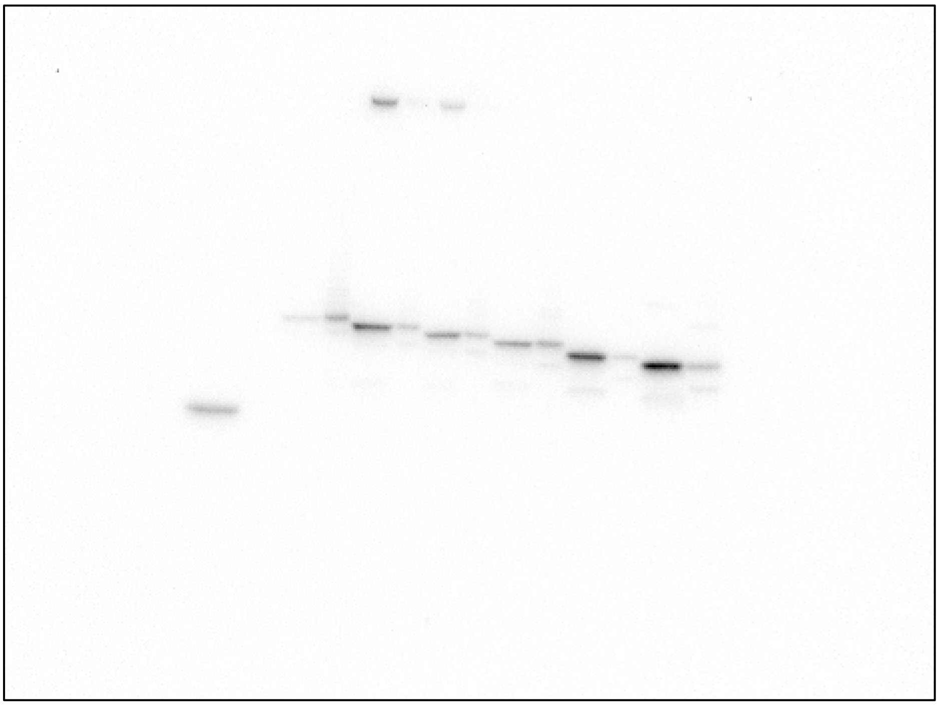

Figure 1d. Tubulin blot

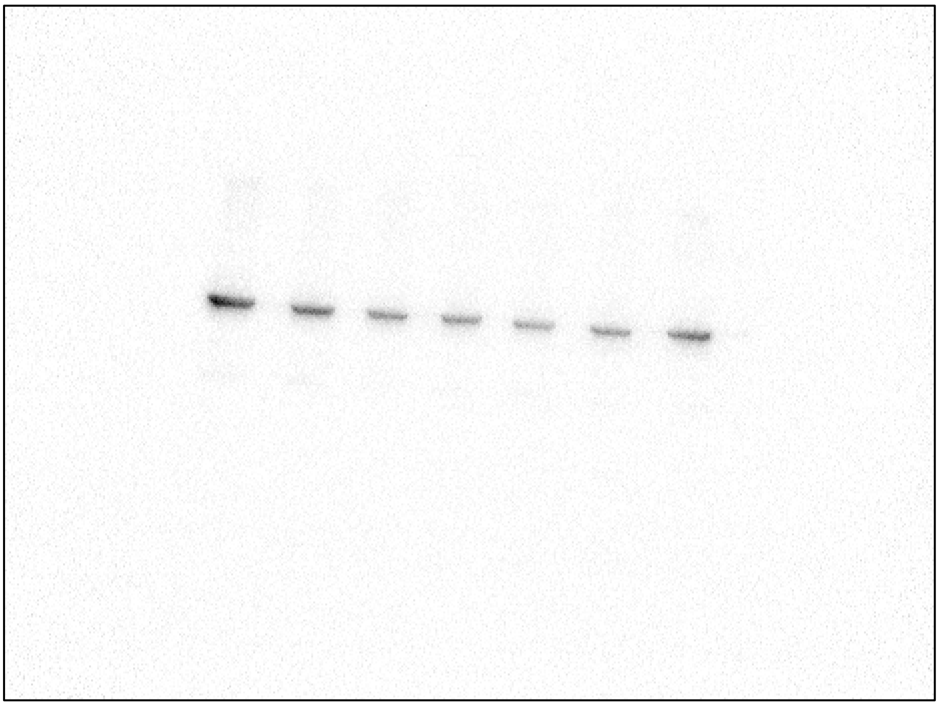

Figure 3d. GFP blot

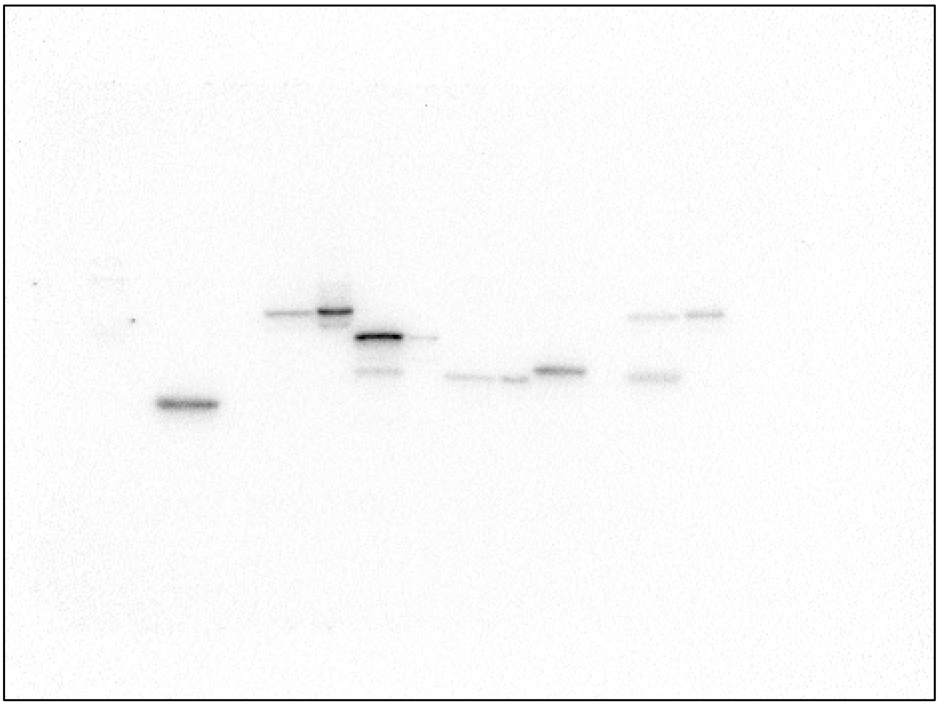

Figure 3d. Tubulin blot

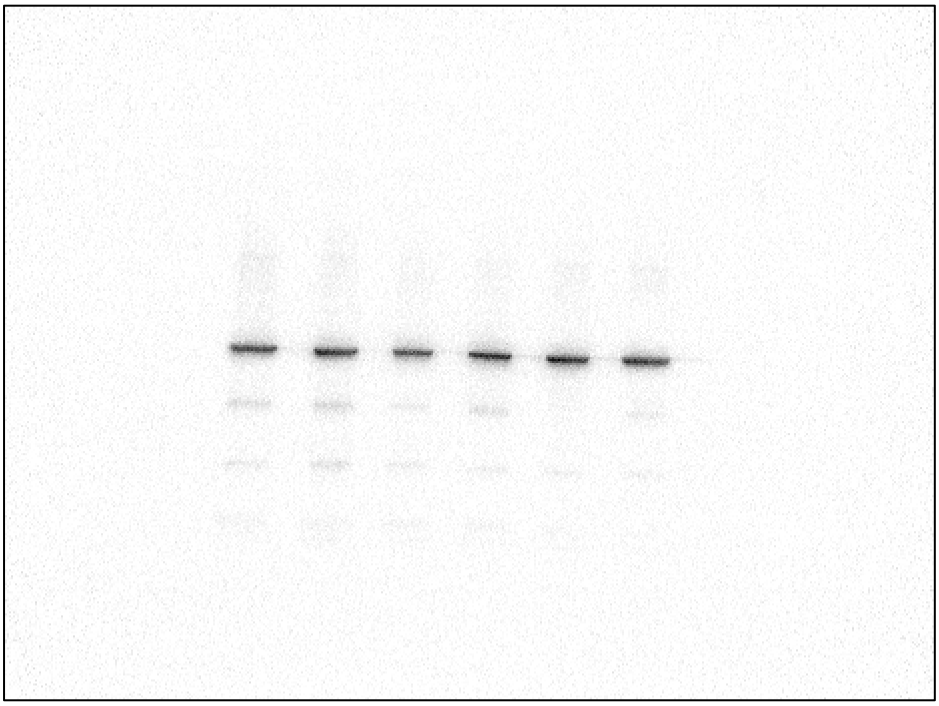

Figure 5e. GFP blot

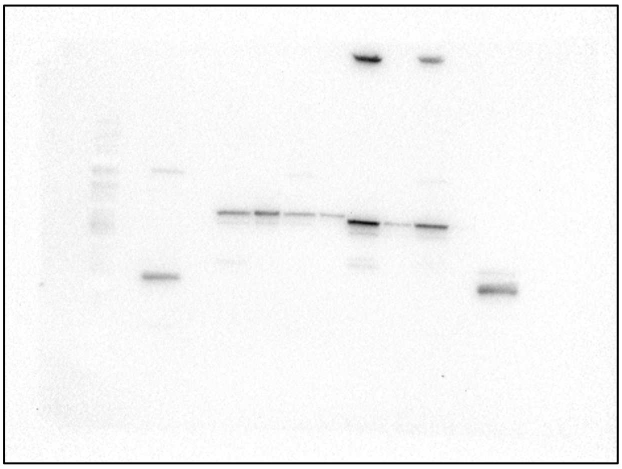

Tubulin blot

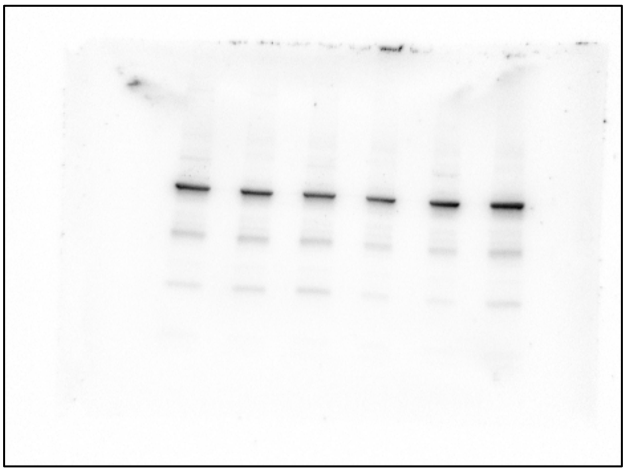

Figure 5e. RFP blot (light)

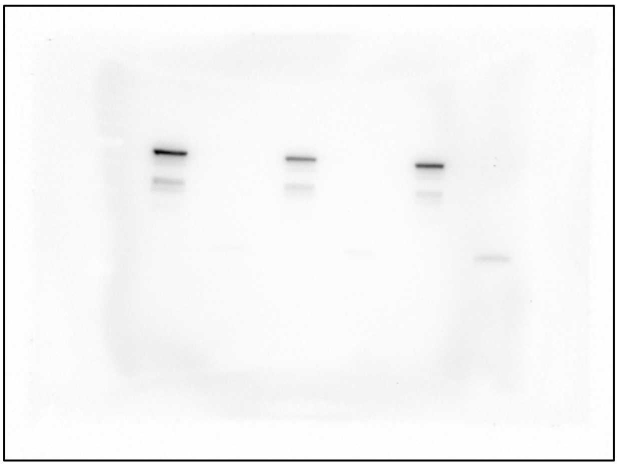

Tubulin blot (heavy)

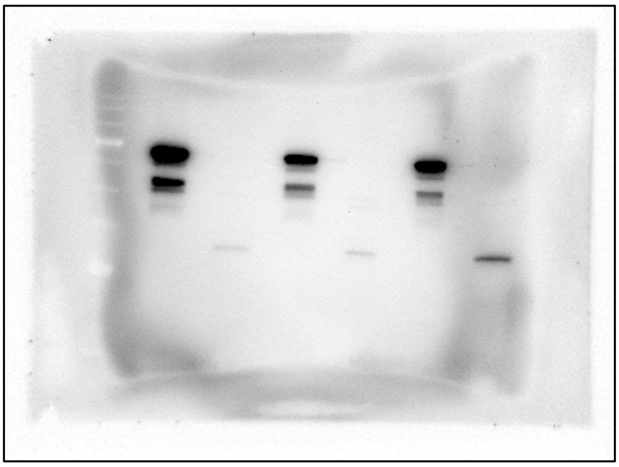

Figure 6d. GFP blot

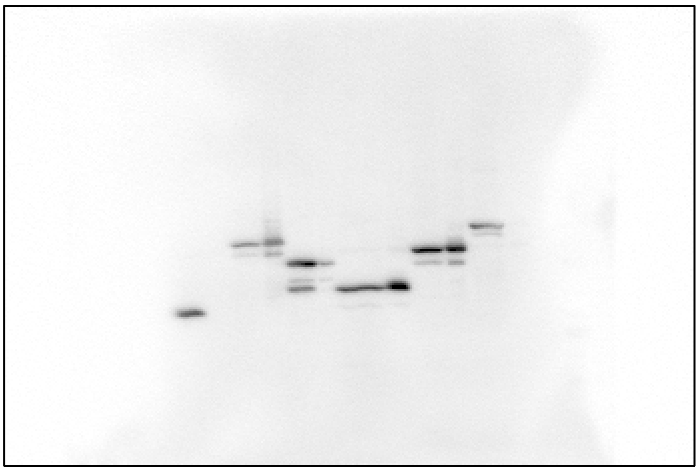

Figure 6d. Tubulin blot

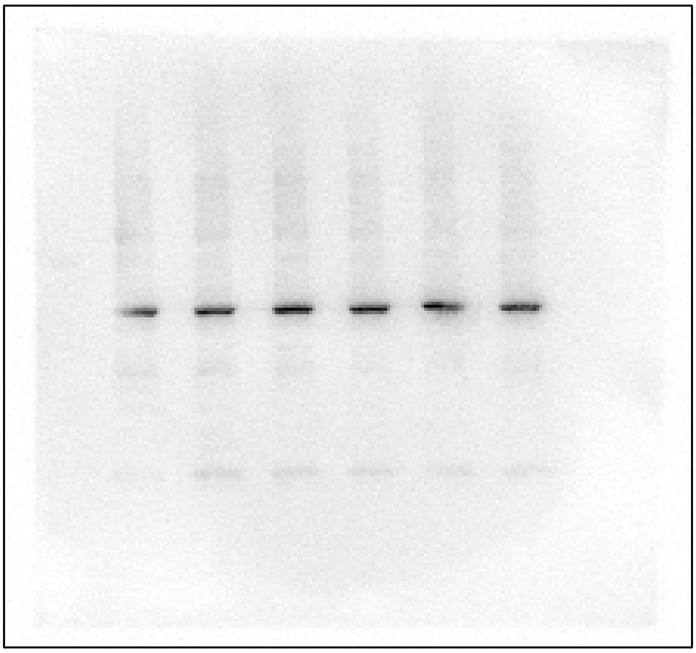

Figure 7f. GFP blot

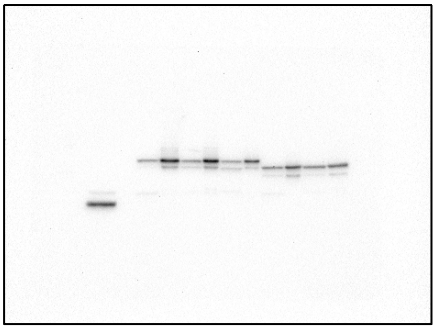

Tubulin blot

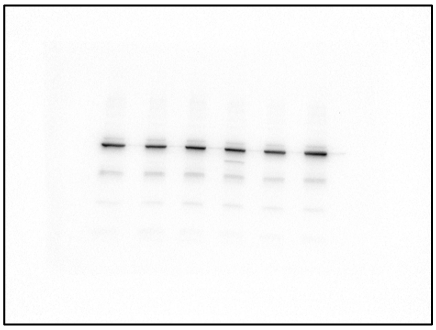

Figure 7f. RFP blot (light)

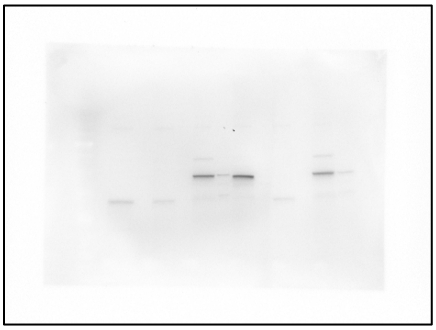

RFP blot (heavy)

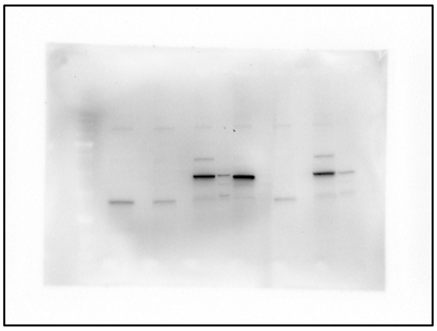

Figure 9b. GFP blot

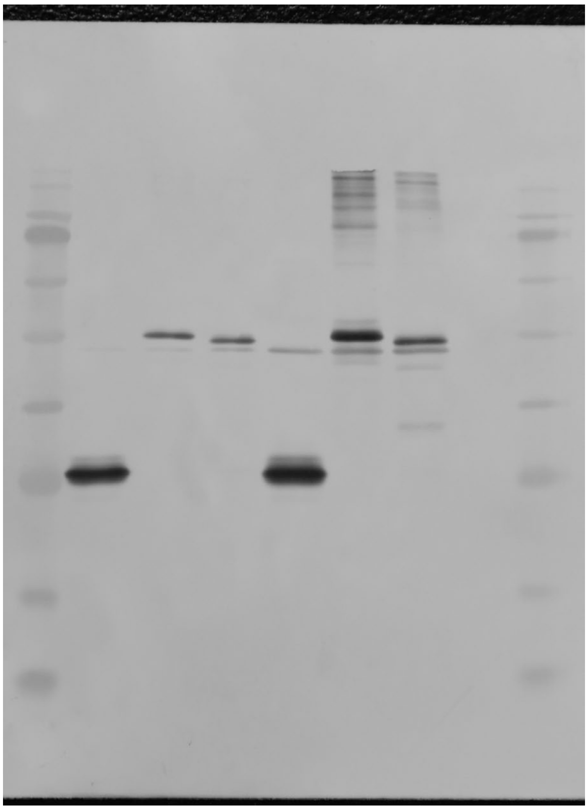

Figure 9b. Tubulin blot

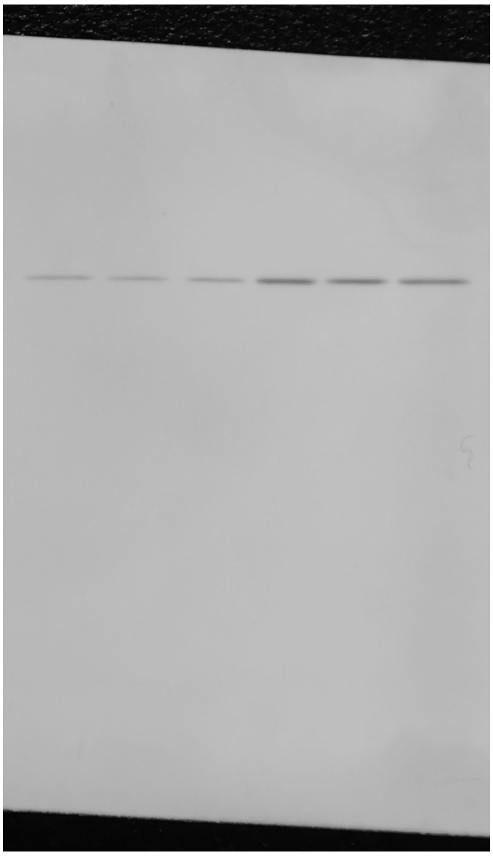

Supplement: Supplementary file 2 — Supplementary Information [file 42003_2024_6410_MOESM2_ESM.pdf]
