# Supplementary material for: Hetero-oligomerization of TDP-43 carboxy-terminal fragments with cellular proteins contributes to proteotoxicity
Source: Commun Biol. 2024 Jun 20;7:743. doi: 10.1038/s42003-024-06410-3 (PMC11190292; doi:10.1038/s42003-024-06410-3)
Supplement: Supplementary file 3 — Description of Additional Supplementary Materials [file 42003_2024_6410_MOESM3_ESM.pdf]

Description of Additional Supplementary Files

File name: Supplementary Data 1

Description: The source data for the main graphs.
